# Detecting When “Quality of Life” Has Been “Enhanced”: Estimating Change in Quality of Life Ratings

**DOI:** 10.4236/ojpp.2013.34A005

**Published:** 2013-11-01

**Authors:** Rochelle E. Tractenberg, Futoshi Yumoto, Paul S. Aisen

**Affiliations:** 1 Departments of Neurology, Biostatistics, Bioinformatics & Biomathematics, and Psychiatry, Georgetown University Medical Center, Washington, D.C., USA; 2 Collaborative for Research on Outcomes and—Metrics; 3 IMPAQ International, Columbia, USA; 4 Department of Neurology, University of California, San Diego, USA

**Keywords:** Longitudinal Analysis, Statistical Method, Scale Type, Data Interpretation

## Abstract

**Objective:**

To demonstrate challenges in the estimation of change in quality of life (QOL).

**Methods:**

Data were taken from a completed clinical trial with negative results. Responses to 13 QOL items were obtained 12 months apart from 258 persons with Alzheimer’s disease (AD) participating in a randomized, placebo-controlled clinical trial with two treatment arms. Two analyses to estimate whether “change” in QOL occurred over 12 months are described. A simple difference (later - earlier) was calculated from total scores (standard approach). A Qualified Change algorithm (novel approach) was applied to each item: differences in ratings were classified as either: improved, worsened, stayed poor, or stayed “positive” (fair, good, excellent). The strengths of evidence supporting a claim that “QOL changed”, derived from the two analyses, were compared by considering plausible alternative explanations for, and interpretations of, results obtained under each approach.

**Results:**

Total score approach: QOL total scores decreased, on average, in the two treatment (both −1.0, *p* < 0.05), but not the placebo (=−0.59, *p* > 0.3) groups. Qualified change approach: Roughly 60% of all change in QOL items was worsening in every arm; 17% - 42% of all subjects experienced change in each item.

**Conclusions:**

Totalling the subjective QOL item ratings collapses over items, and suggests a potentially misleading “overall” level of change (or no change, as in the placebo arm). Leaving the items as individual components of “quality” of life they were intended to capture, and qualifying the direction and amount of change in each, suggests that at least 17% of any group experienced change on every item, with 60% of all observed change being worsening.

**Discussion:**

Summarizing QOL item ratings as a total “score” collapses over the face-valid, multi-dimensional components of the construct “quality of life”. Qualified Change provides robust evidence of changes to QOL or “enhancements of” life quality.

## Introduction

Estimating change in outcomes like quality of life (QOL) is difficult (e.g., Schwartz, 2010; Howard, Mattacola, Howell, & Lattermann, 2011). Thus, it is also difficult to estimate the extent to which any intervention—including “quantification” —can “enhance” life quality. In any case where “measurement” and “measurement of change” are difficult to define or estimate, inferences and conclusions that are based on a simple difference (score at time 1 - score at time 2) may be tenuous, not because the “amount” or “average amount” of change observed is difficult to calculate, but because it is difficult to *interpret* (Tractenberg, Chaterji, & Haramati, 2007). In this manuscript, we describe two methods for estimating change in QOL over one year. In one, changes in QOL ratings are computed and interpreted *at the item level* (Tractenberg, Jin, Patterson, Schneider, Gamst, et al., 2000) without any (inappropriate) measurement assumptions; and in the other, QOL changes are assumed to be measured by the difference in “total QOL scores”. One of these methods, we will argue, is an inappropriate quantification of non-quantitative QOL ratings. The other is an appropriate and interpret-able alternative to understanding if quality of life (as well as other constructs) may have changed, and how.

Kuhn (1996: p. 52) defines the aim of “normal science” to be “…the steady extension of the scope and precision of scientific knowledge”. In the biomedical (mainly clinical) literature there has been a low-level debate going on about the proper role of “psychometric theory” in clinical work. Psychometrics is a scientific domain that focuses generally on measurement principles and procedures, as well as measurement theory building, testing and validation. The output of the field is general for all measurement, but the specific elements tend to be focused on mental, psychological or other difficult-to-measure constructs (e.g., IQ, ability).

As an example of the debate, Fava & Belaise (2005) characterize the importance of “clinimetrics”, defined in 1987 to be “a domain concerned with indexes, rating scales, and other expressions that are used to describe or measure symptoms, physical signs, and other distinctly clinical phenomena in medicine” (Fava & Belaise, 2005: p. 753). Streiner (2003) argues (correctly, in our opinion) that clinimetrics is actually a simple subset of psychometrics. Streiner’s critique of the conceptualization of clinimetrics as a unique and separate domain of inquiry, relative to psychometrics, focuses on the danger posed by clinimetrics’ failure to fully understand or appreciate measurement principles that are fully articulated within psychometrics as a field. Clinimetrics seeks independence from formal and well-established (and burgeoning) knowledge about measurement generally, and some (e.g., de Vet, Terwee, & Bouter, 2003) have argued that the construct of clinimetrics actually serves to heighten appreciation among clinicians and clinical researchers for the critical role that measurement issues play in medicine. This debate, and the risk Streiner identifies, are perhaps especially critical in the current era of “evidence-based” medicine (e.g., Sackett, 1996; but see also de Vet, Terwee, & Bouter, 2003 for the criticality of measurement in evidence-based medicine), as well as in values-based medicine (Fulford, 1989; see also Brown & Brown, 2013). When one is assessing evidence— representing efficacy of an intervention (requiring the precise estimation of change) and balancing this against potential risks, for example—the validity, reliability and precision of the estimates are absolutely essential. “Validity”, “reliability”, and “precision” are all key constructs in psychometrics; the vast majority of biomedical research fails to appropriately define or assess these characteristics of instruments employed to describe symptoms, states, traits or feelings—as well as values and “qualities” (of life, pain, or other patient characteristics). Thus, it is possible to characterize the efforts to integrate “clinimetrics” into biomedical research as fundamentally opposed to Kuhn’s stated aim of normal science (“the steady extension of the scope and precision of scientific knowledge”). It is not possible to characterize clinimetrics as an “anomaly” (p. 52) or “crisis” (p. 69) in Kuhn’s senses, because “clinimetrics” is neither novel nor identifies a violation of any feature that psychometrics has already defined/ addressed.

In this paper we describe a method for the evaluation and determination on which change in QOL has occurred and that is psychometrically sound—and as such, could be utilized appropriately for inferences in clinical research (or other social or economic research involving QOL), as well as in either identifying or prioritizing goals (as in goal directed health care (e.g., Waters & Sierpina, 2006)) or in balancing values in value based medicine (e.g., Brown & Brown, 2013).

While most medical outcomes may be objective, and can easily be quantified, “quality of life” as a goal for treatment, or at least as a consideration in the decision to employ a given intervention, has been increasing over the past 30 years or so (Hand, 2004: pp. 195-6). QOL has become an important outcome in medicine, but its analysis is complicated because it is difficult to show that the differences between any pairs of QOL ratings, over time, for example, are equivalent. That is, subjective ratings cannot always be considered to be true interval scales (i.e., differences between successive levels being equal (Stevens, 1946; De Gruiter & van der Kamp, 2008). This is true for multiple observations within an individual, as well as for multiple individuals, cross-sectionally and longitudinally, complicating efforts to establish responsiveness for items or the instrument overall. This manuscript is focused on the analysis of change in instruments that are constructed using sets of items with patient-reported frequency ratings or with Likert-type (subjective) ratings; these are typically summarized for individuals by summing the item-level ratings, creating a total score.

It might be more appropriate to treat subjective ratings as ordinal rather than interval (Stevens, 1946; Nunnally & Bernstein, 1994); but then summarizing, and drawing inferences based on, ordinal responses could lead to less meaningful results than if the variables were truly interval in nature (Nunnally & Bernstein, 1994; Tractenberg, Jin, Patterson, et al. 2000; Sheskin, 2004). Interpreting differences between individuals, or within individuals over time, is dif-ficult—not to mention invalid and inappropriate—with ordinal measures. In addition, when a total score is computed as a sum of ratings (e.g., frequencies, Likert-scale or other subjective responses) on questions/items representing different *dimensions* of quality of life, the same “total score” can be obtained for a person with a low level of endorsement across most/all items, and for a person with only a few, yet extreme, ratings—and this can be observed for ordinal or interval instruments (Nunnally & Bernstein, 1994; Tractenberg, Jin, Patterson, et al., 2000). That is, whenever items are not exchangeable, whether ratings are absent/present or wrong/right, frequencies, or Likert scales, two identical “total scores” can be obtained by very different patterns of item responses, ratings or endorsements. Thus, difficulty in interpretation of ordinal scores can be compounded by the variability of response patterns that can lead to the same total score; these problems are propagated through an analysis in unpredictable ways when such total scores are used in longitudinal analysis. These features complicate interpretability of total scores, cross-sectionally and especially longitudinally.

Beyond these concerns about the unsupportable assumption that Likert, subjective, or otherwise ordinal (or categorical) ratings can be summed (and this total “score” can be interpreted), a model of how patients’ internal perspectives shift over time (“response shift theory”) has been studied— with different methodologies being suggested to accommodate (or, to be developed to accommodate), the shift in perspectives across patients (see Schwartz, 2010: p. S39; Howard, Mattacola, Howell and Lattermann, 2011). Another approach to understanding change in QOL (or other patient reported outcomes) is to understand the minimally important change (e.g., de Vet et al. 2006)—typically, this must be defined for each instrument—and specifically, “…whether the minimally detectable change of a measurement instrument is sufficiently small to detect minimally important changes” (in the patient’s actual quality of life) (de Vet et al. 2006: p. 54).

Although extensive work has been done to facilitate the sophisticated statistical analysis and modeling of rating scales (see Embretson & Reise, 2000; see also Schwartz et al., 2011), two important factors limit the applicability of these statistical methods to the problem of assessing changes in the quality of life. First, these analytic methods are technical in terms of mathematics and the modeling itself, requiring extensive programming skill or special software (usually both), and in many cases, larger samples are typically found in biomedical research. These methods can be easy to *implement*—but not *interpret*—when instruments are designed for optimal face and construct validity, but were not designed specifically to literally “measure” QOL or change in QOL. Further, many advanced statistical analytic methods might appear useful in the analysis of QOL or change in QOL (see, e.g., Revicki & Cella, 1997; de Vet, Terwee, Ostelo et al., 2006), but these are inappropriate when the investigators have neither built nor validated a theory-driven model of QOL. A second limitation on approaches such as response shift analysis (e.g., Schwartz et al., 2011) is that a generally untestable model of the observed response shift is needed in order to “calibrate” the items and correct for each individual respondent’s internal standard for conceptualization or valuation of the quality of life, or the elements that make up their particular quality of life. Again, without a validated model, the *use* of methods like this one may be straightforward, but *interpreting* it or its results will not be so.

Therefore, although an analysis of change in quality of life *could* be done with some formal analytic methods (e.g., item response theory/latent trait or growth modeling; see Revicki & Cella, 1997; Schwartz et al., 2011), this paper instead focuses on a simple, theoretically-motivated, nonparametric approach to the assessment of changes in QOL over time. The method is demonstrated on QOL ratings given at baseline and one year later by patients with Alzheimer’s disease (AD) participating in a clinical trial (Aisen et al., 2003).

Specifically, many new and complex analytic methods have been published for evaluating QOL and changes in instruments assessing QOL over the past 10 – 15 years. However, the field has not converged on any of these methods as “the best” method to appropriately analyze QOL or change in QOL and yield consistent, reliable, and interpretable results. Because of this lack of “one best method”, the majority of investigators who use QOL or other patient reported outcomes simply administer their instrument of choice and then sum the ratings to obtain a “total score”. This study sought to determine if one-year changes in QOL ratings that are computed and interpreted *at the item level* (Tractenberg, Jin, Patterson et al., 2000) give substantively different evidence that QOL change occurred, as compared to the difference in “total QOL scores”.

## Methods

### Ethics Statement

The data were taken from an NIH-funded multi-center clinical trial (Aisen et al. 2003; ClinicalTrials.gov identifier NCT00004845) that was IRB approved at all participating institutions (see http://clinicaltrials.gov/ct2/show/study/NCT00004845?Term=2783912+[PUBMED-IDS]&rank=1&show_locs=Y#locn for list of study locations at which IRB approval was obtained). The data were obtained deidentified, and were analyzed anonymously.

Two methods were used to compute “change” in QOL over 12 months in the sample. The simple difference approach collapses across items—treating them as exchangeable, summing all ratings at a given administration (baseline, one year), and computes the difference between two observed total scores (e.g., later - earlier). The method called Qualified Change (Tractenberg, Jin, Patterson, et al. 2000; Tractenberg, Gamst, Thomas et al., 2002) examines responses at the two time points for each item, characterizing the type of change they reflect, including “no change” (e.g., improved, worsened, stayed at a low level, stayed at a high level, etc.). The Qualified Change approach organizes and characterizes changes in data at the item level, while the simple difference approach does this at the total-score level, so the interpretations of results could differ depending on the approach.

A key difference between these methods for estimating “change” in QOL over time is that summing item ratings to create a total score assumes the items are exchangeable (i.e., each item represents the construct “QOL” equally well) and interval type (i.e., the difference between any two adjacent ratings is the same as the difference between any other two adjacent ratings—for every item and every individual). While the Qualified Change approach does not assume any given model is operating to account for respondent shifts in calibration, prioritization, or conceptualization of the item or rating levels over time, it does assume that there is no especially reliable information—from any respondent at a single point in time—at the Likert or other subjective rating level (see, e.g., Tractenberg et al., 2007). Instead, in this method, any pair of ratings that remain on one side of the central or “neutral” (e.g., “agree”, “strongly agree”) or “positive” (e.g., “fair”, “good”, “excellent”) value in the scale is assumed to reflect agreement rather than a consistently—reportable level of agreement for any item. In this way, the investigator defines a priori what type and amount of change across the central rating will represent “clinically meaningful change” for any item. Movement across the neutral or center value is then defined as “meaningful” change (qualified as to whether it is a positive shift or negative shift)—but this framework is not provided to the rater.

Collapsing across Likert, ordinal/categorical, or other rating categories compresses rating-level data for an item over time, but this is not conceptualized as a “loss of information” because it is difficult to *justify* the assumption that an individual perceives the rating categories across items—within one test administration—identically. It is impossible to justify this assumption across time or across individuals (which is one unarticulated rationale behind response shift analysis). Instead of assuming that we can build a model that correctly predicts how respondent perspectives change over time (response shift), or that respondents consistently use the same perspectives to rate every item over time, the Qualified Change method only assumes that a “real” change is represented by change over time from a rating on the “agree”, “neutral”, or “disagree” sections of the scale to any of the other sections. In the Qualified Change approach to the calculation of change, the amount of change is not assumed to be estimable—only when and that evidence of change has been observed.

This study therefore compared the support provided by each method to substantiate an inference that “change has occurred”. This evidence comparison is not probabilistic, but can still be informative about the level of support for a conclusion of “change” derived from each method. This study examines exactly how the results from the two approaches differ in terms of the evidentiary weight derived from each method.

### Subjects

258 Alzheimer’s disease patients in a clinical trial of non-steroidal anti-inflammatory agents responded to 13 QOL items at two visits 12 months apart. The study has been described previously (Aisen et al., 2003), and compared two agents (rofe-coxib (Rof), naproxen (Nap)) to a placebo. QOL was one instrument out of the large set that was administered in this multi-center clinical trial, which had a null result. These analyses focus only on the QOL responses and ignore any treatment effects (since none were reported in the original intent-to-treat analysis).

### Instrument

QOL-AD consists of 13 items that are rated—with respect to the patient’s QOL—by both the patient and the caregiver (Logsdon, Gibbons, McCurry, & Teri, 1999; Logsdon, Gib bons, McCurry et al., 2004), as being “poor”, “fair”, “good” or “excellent”. The standard scoring algorithm is to sum the ratings on the items from each respondent (patient or caregiver). In our analyses we used only the ratings obtained from the patient. The instrument was administered at the baseline and 12-month visit in the study.

### Data Analysis

#### Simple difference, “change in overall QOL”

The ratings on each of the 13 items on the instrument are assigned numeric values (“poor” = 1, “fair” = 2, “good” = 3, “excellent” = 4) so that high ratings reflected good QOL and low ratings reflected poor QOL. As implied by the “scoring”, the total “QOL score” reflects overall level of QOL. To evaluate change in overall QOL using this instrument (and many others), the total score at the baseline visit was subtracted from the same individual’s 12 month visit.

#### “Qualified change in QOL”

The Qualified Change approach to the assessment of “meaningful” change over time has been described and developed elsewhere (Tractenberg, Jin, Patterson et al., 2000; Tractenberg, Gamst, Thomas et al., 2002). Briefly, this method for assessing change was developed specifically for instruments made up of *non-exchangeable items* in a longitudinal framework. In the Qualified Change approach each item is subjected to the following calculus (originally described in *Change* (Tractenberg, Jin, Patterson et al., 2000): *Before* computing differences between actual item ratings, the investigators classify all possible differences, based on the difference and the starting and ending values. *A priori*, the difference values are classified according to whether (given the start and endpoints) the difference represents worsening, improvement, no change-staying “good”, no change-staying “bad” (see Table 1). After this classification scheme is developed, then the difference between ratings given on two occasions is computed/characterized for each item and individual.

Table 1 shows the scheme used in this analysis to classify the types of “qualified” change in the QOL items we analyzed. The item-level analysis then focuses on the modal response-change type(s), which reflects the tendencies of the group to exhibit the types and directions of change of greatest clinical importance (established *a priori*).

Multiple modes are possible; the results are reported not as a single-value quantitative summary (e.g., mean change in total score) but as frequencies of each type of change. Because the change types are *countable*, this *is* a quantification of “change in QOL”.

#### Evidence of change

We evaluate the evidence, obtained under each analytic method, in the data supporting a claim that “change in QOL” had occurred over the 12 month study (see Mislevy, 2003 for full discussion of evidence to support claims of change in assessment). Support for the claim of “change in QOL” can be undermined by plausible alternative explanations that might be supported by rebuttal data, but the reasonableness of alternatives must also be evaluated. In this study, the change values under each method were the data used to support the same claim, namely, “change in QOL has occurred over the 12 month interval”. Plausible alternative explanations for the data from each method (i.e., other reasons for observing what we would hope to *interpret* as “change in QOL” than actual change in QOL) were generated. The “comparison” of the two methods is therefore a comparison of the strength of the support that each is showing “*true* change in QOL”, relative to the strength of the plausible alternative explanations for the claim of “*apparent* change in QOL”. A method with results that are *sufficient* to overcome any plausible alternative explanation is deemed “supporting the claim that change in QOL <truly> occurred”. A method with results that are *insufficient* to overcome a plausible alternative explanation for the change that was observed is deemed to be in need of further data. Methods that are insufficient to overcome the alternatives do not support valid inferences.

#### Simple difference, “change in overall QOL”

The total “QOL score” reflects overall level of QOL. The results are reported as a single-value quantitative summary (e.g., mean change in overall QOL). This scoring, and the use of a total score, is consistent with the original (Logsdon, Gibbons, McCurry, & Teri, 1999) as well as with subsequent analyses (Logsdon, Gibbons, McCurry et al., 2004) of QOL data over time. The following assumptions are required to support a claim of *change in QOL on the basis of simple differences*:

- The sum of the QOL item ratings reflects an overall level of QOL for the person.
- If the overall level of QOL for a person changes, then their total QOL score will change.
- Changes in QOL items that do not result in a change in the total QOL (because they offset each other), lead *correctly* to the conclusion that overall, the person’s QOL has not changed.

These assumptions reflect the *implicit* rationale(s) for basing a decision about change—i.e., deciding that it has happened—on change in total score over time. This is true for claims about change that are based on *any* measure of QOL, as well as most other instruments across clinical and biomedical research where clinician-—and patient-reported outcomes are used.

#### Qualified change in QOL

The Qualified Change approach classifies each individual’s observed change (type) on each item. The group is summarized in terms of the types of change observed on each item. The results are reported *not* as a single-value quantitative summary, but as a frequency table that quantifies results for each item. One particular type of change can be emphasized (e.g., summarizing and/or comparing the proportion of two groups reporting “stable-bad” on each item) according to the investigators’ interest (e.g., Tractenberg et al., 2002; Tractenberg et al, 2007).

#### The following assumptions are required to support a claim of Qualified Change in QOL

- Each QOL item has the potential to contribute unique information to our conceptualization of a person’s overall QOL (i.e., items are not exchangeable).
- Change should not be mistaken for a failure to give the same answer at two opportunities.
- Different types of change, and different ways of reflecting no change (i.e., stability that is positive, neutral, or negative) can contribute unique information to our conceptualization of change in a person’s QOL.
- These types of change and stability can inform our perspective of the individual’s change in QOL.

These assumptions reflect the *explicit* rationale for using Qualified Change in the determination of whether (clinically) meaningful change has occurred when using an instrument comprising items that are not exchangeable.

The main difference between this approach and the simple-difference approach is that under the Qualified Change approach we define “change in *QOL” a priori* to represent the essential “message” of the differences between any two ratings over time, and independent of the tendency of a respondent to select the same, or different, rating levels at any two times without intending to represent a real change. This is a fundamental feature of a Qualified Change—it is not simply the difference, but is a qualitative evaluation of change—in some cases, defined as the minimum amount of change deemed “clinically relevant”. When QOL ratings are separated over 12 months, as in the present case, it is particularly important to avoid concluding that “change” has occurred when the respondent simply failed to choose the same rating as they did previously (i.e., measurement error).

Collapsing across all possible categories of agreement or disagreement will result in the loss of this rating-level data, but there is *no reason* to expect that there is relevant data at this level—because in fact, these instruments are rarely created according to psychometric or measurement principles, even in the case (like this one) where the test was intended by its crea-tor(s) to be summarized as a total score (Logsdon, Gibbons, McCurry, & Teri, 1999). More importantly, there may be little reason (and/or insufficient reluctance) to assume that incremental changes (e.g., strongly agree to agree) are error-free. Instead, under the Qualified Change method, any positive classification of observed change in rating (“improved”) is only assumed to reflect “some improvement in QOL occurred” rather than to reflect a consistently-reportable *level* of improvement on the given QOL item. It may be argued that collapsing over difference-values “eliminates information”, but that argument assumes there is reliable and subject-independent information to be obtained. The Qualified Change method does not make this assumption, and may reduce measurement error as well as errors in inference that are based on untested and improbable assumptions. Therefore the differences between *categories of change* (improvement, worsening, or no change) can now be treated as equivalent in the sense that these differences might be more reliably distinguished than differences between the specific ratings themselves.

These two methods are different in terms of the approach to, and interpretation of, “change”. The differences are reflected in the data (total scores vs. items), and in the warrant and backing for the argument that “change in QOL has occurred”. The purpose of this study was to compare the weight of evidence derived under each method, not to compare the methods themselves, since the two methods are not, in fact, strictly comparable.

## Results

### Total score approach results

Loss of QOL was observed within one treatment arm (*p* < 0.05) but the second treatment arm had equal loss but greater variability so that loss in QOL total scores over one year was not significantly different from zero (*p* > 0.08); individuals in **both** active arms lost an average of 1.2 points in total QOL. Total QOL loss for the placebo arm was 0.64 point (*p* > 0.3) over the 12 month study period. A one-way ANOVA showed that overall, 1-year change in total QOL “scores” was not significantly different across groups (F(2, 255) = 0.185, *p* = 0.83).

### Plausible alternative explanations for observed differences in total score (data)

Any difference in total score may be simply a function of a respondent intending, but failing, to respond with the same rating provided at the first visit. It is possible that the construct “QOL” is inherently unstable and so it is not the person’s QOL that has changed but rather, QOL has a different meaning at different visits. Similarly, it is possible that the respondents are interpreting the QOL items differently over time, so that what is characterized as “change” by a simple-difference computation instead reflects unreliability of the instrument. Finally, it is possible that, rather than reflecting real change, observed differences in total scores represent spurious variations in responses that might be expected from an instrument with lower reliability—or that the application of arithmetic functions (addition of ratings and subtraction of totals) that are inappropriate for ordinal responses resulted in spurious (the appearance of) change.

### Qualified Change approach results

Roughly 60% of all change in QOL items was worsening in each arm; among items where both worsening and improving occurred, the proportion worsening exceeded improving for *one* item in each arm: marriage (Rof), friends (Nap), and living situation (Pla). For other items, worsening and improving accounted for roughly 50% (each) of all changes observed (i.e., equal amounts of improvement and worsening occurred). For each item, 17% - 42% of all subjects experienced change (whether worsening or improving). To explore the item-level changes, t-tests comparing the differences over time on item ratings to the test value of zero (i.e., not qualified change) found that family, marriage, whole self, chores, and whole life ratings were significantly lower at the second visit than the first; these results were significant within one treatment arm only (Rof).

### Plausible alternative explanations for observed Qualified Change

The classifications of change types may reflect spurious changes, and not “real” change—even though the method was intended to limit this, it is still a possible explanation for the observed differences. It is also possible that the construct “QOL” is inherently unstable, and if so, then the variability in ratings given over time would need to exceed the threshold established in the pre-analysis qualifying of change types (Table 1). This is a testable hypothesis, and it also highlights the purpose of qualifying the level of change the investigator require before you conclude that change has actually happened. However, the plausibility of this alternative requires that all change classifications have been affected similarly; this is challenging to test since the Qualified Change method assumes the items are *not* exchangeable.

## Discussion and Conclusions

First, The Qualified Change method was developed to avoid the case where a total-score approach to change can obscure changes in non-exchangeable items that may be changing over time in ways that cancel out other items, reducing *apparent* change in the total score (e.g., overall QOL). In this study, we determined *a priori* that one-point differences over time would be treated as “no change” (see also Tractenberg et al., 2000; Tractenberg et al., 2007). This essential feature of the Qualified Change approach reduces the likelihood of falsely concluding that change has happened. The total score/simple difference approach assumes that any (one-point) change on an item is “real change” and not simply a failure to give an identical second report. However, the threshold for labeling differences in ratings over time as “change” can vary depending on the instrument, or the context. For example, we have treated a one-point change, when it indicated worsening, as “real change”, while treating a one-point change, when it *might* indicate improvement, as “no change” (Tractenberg, Gamst, Thomas et al., 2002) in the context of a behavioural problem intervention. This reflected our desire in that analysis to detect *any* evidence of worsening, and to be less sensitive to spurious evidence of “improvement”.

Recent attention has been given to the formal measurement properties of QOL assessments. In their survey of QOL instruments for use in dementia (specifically), Ettema, Dröes, de Lange, Mellenbergh and Ribbe (2005) reported that two of six published QOL instruments reported some level of responsiveness to “change”, but the empirical definition (or validation) of that “change” to which the QOL instruments were detecting was not specified. Terwee, Dekker, Wiersinga, Prummel and Bossuyt (2003) note that “responsiveness” of QOL instruments is not a “separate measurement property” from validity, while Frei, Svarin, Steurer-Stey and Puhan (2009) include documentation of the instrument’s responsiveness in the “validation” step of their five-step outline for the development and validation of self-efficacy instrumentation. The Qualified Change approach to analyzing change over time was not intended to represent “responsiveness” nor treatment effects; instead it was intended to facilitate the definition of “clinically important change” when the data are ordinal, but subjectively ranked. Importantly, the qualification of change is done before the data are analyzed, so this is clearly not a method for *detecting* change, but for identifying what would constitute “change” if it was observed—and then determining the evidence that this has indeed been observed (and in whom). This method can therefore be used for the definition (estimation) of responsiveness in QOL or self-efficacy instruments, as well as other contexts (including behaviour, e.g. Tractenberg et al., 2002; and classroom attitudes, e.g., Tractenberg et al., 2007). It can also be incorporated appropriately into goal-directed health care as well as value-based medicine and value based medical analyses. For example, the simple-difference approach to incorporating change in QOL into goal-directed health care would be to specify a target number of “points” to gain (or zero lost) on a QOL total score. However, as demonstrated in our analysis, points may (or may not) be gained or lost in the total score while obscuring otherwise meaningful change or stability. Thus, it is possible to conceptualize using Qualified Change to define more specific (to the patient) goals relating to particular QOL dimensions (to improve or maintain) as compared to a simple-difference approach to goal setting.

The Qualified Change method was specifically designed for cases where items are non-exchangeable and any two people can derive the same total score in very different ways at the item level—making it applicable to many patient-reported outcome contexts. Overall, two individuals may appear to have the same “level” of QOL, but one may have many items with low ratings while another may have very few items with high ratings and the rest with very low ratings. These two cases represent very different types of respondents, but their total scores are identical. If the same individual fits these two patterns at successive visits, their total score will “show” no change, but the pattern of item responses shows a clear shift for this individual. An item-level approach to the change that can be reflected in instruments such as QOL can highlight differences that are obscured in total-score analysis (as in other instruments, see Tractenberg, Gamst, Thomas et al., 2002).

When the item-level changes are summarized and presented (rather than summarized with a single value), the plausibility of alternative explanations for results can be considered, as we have presented here. For example, the simple difference results suggested little loss in QOL total score whereas Qualified Change results suggested that at least 17% of any group experienced change on every item, with 60% of all change being worsening. This highlights the fact that a simple difference treats items, and item ratings that increase and decrease by similar amounts, as *exchangeable*. Similarly, two small decreases will offset (be treated as exchangeable with) one slightly larger increase, resulting in apparently no change in overall QOL. These examples highlight the consistency of a Qualified Change approach, and the *inconsistency* of a simple difference approach, with determining if goals have been achieved in a goal-directed health care context.

This manuscript has focused on a key failure by biomedical researchers making inferences about QOL/changes in QOL to appreciate the critical—fundamental and foundational—mea-surement principles associated with attempts to assess the construct of “quality of life”, or changes in this construct within person/over time. In his 2004 book, “*Measurement theory and practice: The world through quantification*”, David Hand includes one chapter on measurement in medicine. Summarizing his brief review of the varieties of definitions of “quality of life”—which ranges from Bentham (“well being”, 1834: p. 76) to the World Health Organization (“health related quality of life”, WHOQOL Group 1993), Hand articulates that “…the one thing that most researchers would agree on is that quality of life is a multidimensional concept” (p. 196). However, Hand goes on to articulate that “…if we are to be able to use the concept (of QOL) to make overall social or economic decisions, it is necessary to reduce it (QOL) to one dimension—essentially using a pragmatic (or, one might say in this context, clinimetric) approach” (p. 196). This statement suggests that Streiner (2003) is *correct*; and de Vet, Terwee & Bouter (2003) were *incorrect* in their assessment that clinimetrics serves a useful purpose by bringing attention to measurement challenges in medical research or medicine. That is, if most researchers agree that QOL is multidimensional (Hand, 2004: p. 196), then how can it be fruitfully utilized when collapsed—without any consideration of its fundamental measurement properties—into a single dimension?

The traditional simple difference approach to summarizing change in QOL (and constructs like it) does collapse the typically multi-dimensional QOL instrument into a single value summary. In so doing, this approach to estimating change also relies on a key assumption that may not be particularly supportable. The assumption is that any person responding “excel-lent”/𠇜strongly agree” at the first assessment and “good”/ “agree” at the second assessment is accurately reporting his/her life quality or agreement with an item at both times. That is, it assumes any observed difference in ratings to the same item accurately reflects a real change in the rater’s level of the construct. It is not possible to determine the validity of this assumption, or whether a person tried, but failed, to report the same level as in the previous rating. The assumption is strict, and may not be entirely appropriate with subjective responses. That is, an investigator may feel that observing a report of “good” at time 1 and “fair” at time 2 does not necessarily mean that a decrease in quality of life has occurred; if QOL has not actually worsened on this item, then even nonparametric alternatives are not appropriate to overcome such variation. However, once this rule (i.e., that “good” at time 1 roughly equals “fair” at time 2) is established *a priori*, qualitative responses can be systematically characterized and then *counted*. These *counts* then have the interval properties (Stevens, 1946) requisite for parametric or nonparametric statistical analysis.

The Qualified Change method can be used to collapse several “small” changes into broader change categories, and so may be made more or less conservative (obscuring more subtle changes) or more sensitive to one type of change (e.g., worsening) than another (e.g., improvement). Such flexibility, as shown in Table 1, is a feature of this approach to interpreting item level change in subjective constructs. This approach is widely applicable—not simply in biomedical or clinical contexts but in any situation where subjective judgements, particularly across multiple dimensions, are being elicited across respondents and over time.

Unreliability in an instrument cannot be entirely circumvented by either the traditional simple-difference or Qualified Change approach; but the alternative interpretations for the Qualified Change results are generally *weaker* than the alternatives to the simple differences. Any item-level analysis will clarify what the total score summarizes, but our assessment of the plausible alternative explanations for “observed changes” in QOL suggests that more and stronger evidence of change was obtained in a Qualified Change approach, which reflects change explicitly and contemplates *a priori* alternative explanations for observing change (or apparent stability). Reports or characterizations of the responsiveness of an instrument (e.g., Garcia, Cella, Clauser et al., 2007; Meyer & Clayton, 2009; Rothman, Burke, Erickson et al., 2009) should be fully consistent with principles of measurement, and should include consideration of whether the planned analysis will collapse over multiple, salient, dimensions as well as over differentially-responsive items. Careful consideration of measurement principles permits researchers (and health care providers) who use subjective ratings to further the aims of “normal science” as defined by Kuhn (1996: p. 52).

## Figures and Tables

**Table 1:** Qualified change: possible change for any given item from time 1 (baseline) to time 2 (12 months later)—and its characterization.

Time 1	Time 2	Change type
POOR	POOR	Stable-bad
FAIR	POOR	Δ−
GOOD	POOR	Δ−
EXCELLENT	POOR	Δ−
POOR	FAIR	Stable-bad
FAIR	FAIR	Stable-good
GOOD	FAIR	Stable-good
EXCELLENT	FAIR	Stable-good
POOR	GOOD	Δ+
FAIR	GOOD	Stable-good
GOOD	GOOD	Stable-good
EXCELLENT	GOOD	Stable-good
POOR	EXCELLENT	Δ+
FAIR	EXCELLENT	Δ+
GOOD	EXCELLENT	Stable-good
EXCELLENT	EXCELLENT	Stable-good

Δ+: change towards better QOL (at least **two** levels); Δ−: change towards worse QOL (at least **one** level). Stable-good: rating is FAIR, GOOD or EXCELLENT at both visits or is rated one level better than FAIR or GOOD; Stable-bad: rating is POOR at both visits or is rated POOR initially then FAIR subsequently. This qualification scheme emphasizes sensitivity to any worsening, requiring greater evidence to support the conclusion of “any improvement”.
